# *C. elegans* flavin-containing monooxygenase-4 is essential for osmoregulation in hypotonic stress

**DOI:** 10.1242/bio.017400

**Published:** 2016-04-27

**Authors:** Nisha Hirani, Marcel Westenberg, Paul T. Seed, Mark I. R. Petalcorin, Colin T. Dolphin

**Affiliations:** 1 Institute of Pharmaceutical Science, King's College London, Franklin-Wilkins Building, 150 Stamford Street, London SE1 9NH, UK; 2 Division of Women's Health, King's College London, St Thomas’ Hospital, London SE1 7EH, UK

**Keywords:** Flavin-containing monooxygenase, FMO-4, *C. elegans*, Hypotonicity, Osmoregulation

## Abstract

Studies in *Caenorhabditis* *elegans* have revealed osmoregulatory systems engaged when worms experience hypertonic conditions, but less is known about measures employed when faced with hypotonic stress. Inactivation of *fmo-4*, which encodes flavin-containing monooxygenase-4, results in dramatic hypoosmotic hypersensitivity; worms are unable to prevent overwhelming water influx and swell rapidly, finally rupturing due to high internal hydrostatic pressure. *fmo-4* is expressed prominently in hypodermis, duct and pore cells but is excluded from the excretory cell. Thus, FMO-4 plays a crucial osmoregulatory role by promoting clearance of excess water that enters during hypotonicity, perhaps by synthesizing an osmolyte that acts to establish an osmotic gradient from excretory cell to duct and pore cells. *C. elegans* FMO-4 contains a C-terminal extension conserved in all nematode FMO-4s. The coincidently numbered human FMO4 also contains an extended C-terminus with features similar to those of FMO-4. Although these shared sequence characteristics suggest potential orthology, human *FMO4* was unable to rescue the *fmo-4* osmoregulatory defect. Intriguingly, however, mammalian *FMO4* is expressed predominantly in the kidney – an appropriate site if it too is, or once was, involved in osmoregulation.

## Abstract (precis)

**Summary:** *C*. *elegans* lacking *fmo-4* fail to clear body water following hypotonic exposure resulting in rapid swelling and rupture, indicating that FMO-4 is required in the hypoosmotic stress response.

## INTRODUCTION

Alterations in cell and systemic osmolarity can have serious physiological consequences and the majority of animal species employ diverse osmoregulatory mechanisms to control water homeostasis (Hohmann et al., 2007; Hoffmann et al., 2009; Wood, 2011). At the cellular level changes in extracellular tonicity will drive water flux across the plasma membrane resulting in either cell swelling or shrinkage and consequent perturbations in intracellular osmolarity. To detect and readjust altered volume cells employ an array of sensory and effector systems that mediate either regulatory volume decrease (RVD) or regulatory volume increase in response to cell swelling and shrinkage, respectively (Lang, 2007). Nematodes, whether free-living or parasitic, require aqueous environments for survival resulting in exposure to often challenging osmotic conditions that may be subject to rapid change, for example following rainfall. A permeable cuticle and pseudocoelomic cavity under hydrostatic pressure complicates these osmoregulatory challenges (Wharton and Perry, 2011). In its natural habitat of plant-derived organic material (Chen et al., 2006) the nematode *Caenorhabditis elegans* will frequently be exposed to differing osmotic microenvironments and has evolved a complex osmoregulatory armoury that is slowly being experimentally revealed (Choe, 2013). Important sites for water homeostasis in *C. elegans* include intestine, hypodermis and, in particular, the excretory system and a number of significant osmoregulatory genes are expressed in one or more of these tissues (Choe, 2013).

To date, studies in *C. elegans* have focused primarily on identifying sensory, behavioural and recovery mechanisms employed during hypertonic stress. Worms exposed to conditions of hypertonicity may shrink dramatically with associated loss of mobility but can recover body volume and movement within a few hours (Lamitina et al., 2004). Recovery is associated with accumulation of glycerol (Lamitina et al., 2004) and regulation by WNK-1 (with-no-lysine) and GCK-3 (germinal centre kinase-3) serine-threonine protein kinases (Choe and Strange, 2007). In contrast, when exposed to water *C. elegans* swells gradually with reduced mobility, due to increased hydrostatic pressure following inward water flux, but recover when returned to normal osmotic conditions (Huang et al., 2005). The relative lack of research into mechanisms enabling *C. elegans* to endure and recover following sudden hypotonic exposure may be, in part, because hypoosmotic sensitivity phenotypes are observed relatively frequently often with genes that likely act indirectly rather than directly affecting a hypotonicity-related RVD pathway. For example, a number of genes associated with cuticle formation and stability, e.g. *acs-20* (Kage-Nakadai et al., 2010), *cuti-1* (Fritz and Behm, 2009) and *tsp-15* (Moribe et al., 2004), exhibit hypoosmotic sensitivity presumably because cuticle integrity and permeability is compromised leading to water influx and increased turgor pressure. These indirect effects can complicate functional dissection of dedicated hypotonicity-related RVD mechanisms.

Flavin-containing monooxygenases (FMOs) comprise a large family of NADPH-, FAD- and O_2_-dependent enzymes active at heteroatom centres, particularly S and N, in structurally diverse compounds (Ziegler, 2002; Krueger and Williams, 2005). In mammals five distinct functional FMOs, numbered 1 to 5, exist each of which is encoded by a discrete gene (Phillips et al., 1995; Hernandez et al., 2004). Although classified as xenobiotic-metabolizing enzymes (XMEs) this may be a misnomer as FMOs with physiological or biocatalytic roles have now been described, e.g. in intracellular redox regulation in *Saccharomyces* *cerevisiae* (Suh et al., 1999), glucosinolate biosynthesis in plants (Schlaich, 2007) and alkaloid-based chemical defence in insects (Sehlmeyer et al., 2010). Very recently, *C. elegans fmo-2* has been demonstrated to promote health and life span via a HIF-1 pathway (Leiser et al., 2015). Mammalian *FMOs* also appear to have endogenous functions as recent reports have revealed that *FMO1* and *FMO5* play roles in the regulation of energy homeostasis and metabolic ageing, respectively (Veeravalli et al., 2014; Gonzalez-Malagon et al., 2015). Although human *FMO3* null homozygosity results in trimethylaminuria (Dolphin et al., 1997) there is no other apparent organic disease (Mitchell and Smith, 2001) suggesting it does not play any significant endogenous function. However, *FMO3* has recently been associated, both directly and indirectly, with a range of conditions, including atherosclerosis, cholesterol balance and glucose and lipid metabolism indicating it may actually be a rather important modifier of human health and disease (Schugar and Brown, 2015). Thus, FMOs should no longer be defined exclusively in terms of xenobiotic detoxification but rather as enzymes with a wide functional repertoire including specific endogenous biological roles.

We previously identified and described the *fmo* family of *C. elegans*, compared them with orthologous genes present in *C. briggsae* and reported that preliminary examination of *C. elegans fmo* deletion strains revealed that, while strains with non-functional *fmo*-*1*, *2*, *3* or *5* are apparently phenotypically normal, the *fmo-4*^−/−^ strain RB562 [*fmo-4*(*ok294*)] displayed hypoosmotic sensitivity (Petalcorin et al., 2005). We have reexamined this in detail and confirm *fmo-4*(*ok294*) exhibits a hypoosmotic hypersensitivity phenotype as following transfer to water worms swell rapidly resulting, in many case, in violent rupture. We confirm that *fmo-4* is hypodermally expressed (Petalcorin et al., 2005) but report that, in addition, *fmo-4* is also highly expressed in duct and pore cells. Thus, FMO-4 appears to function as an osmoregulatory brake to attenuate excessive body fluid accumulation during sudden hypotonic exposure. Although the precise underlying FMO-4-mediated role has not been identified we discuss possible related enzymatic activities and osmoregulatory mechanisms.

In comparison to other nematode FMOs, FMO-4 has a C-terminal extension that contains predicted membrane spanning domains and terminates in a conserved consensus sequence. Interestingly, mammalian FMO4 (numbering is purely coincidental) is also extended at the C-terminus with predicted structural and sequence features that, particularly in Eutherians, are intriguingly similar to those in the C-terminal extension of FMO-4. We suggest these marked similarities represent evidence that nematode *fmo-4* and mammalian *FMO4* evolved from a common, albeit ancient ancestor. Although human FMO4 was not able to rescue RB562, suggesting divergence of catalytic activity with FMO-4, human FMO4 mRNA is abundant in the kidney – an appropriate site of expression if mammalian FMO4 has, or once had, a role in osmotic homeostasis.

## RESULTS

### Loss of *fmo-4* results in severe hypoosmotic sensitivity

**Fig. 1.**
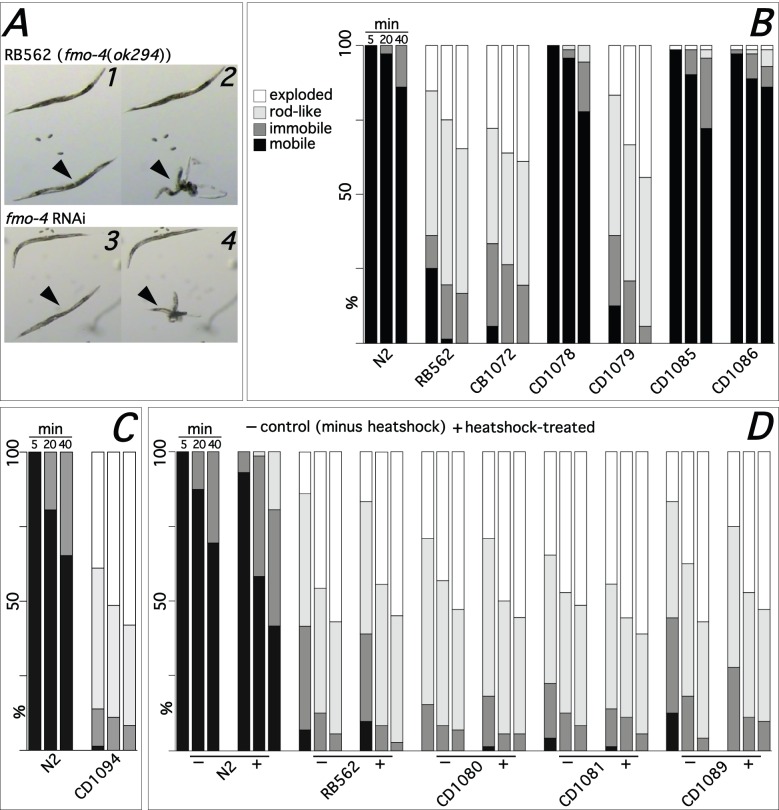
***fmo-4*(*ok294*) hypoosmotic sensitivity and rescue assay.** Following transfer to distilled water adult RB562 [*fmo-4*(*ok294*)] (A1,A2) or *fmo-4* RNAi-treated N2 (A3,A4) worms rapidly stop thrashing, becoming first immobile then rod-like (arrowheads, A1,A3) and finally, in the majority of individuals, undergoing explosive evisceration (arrowheads, A2,A4). The ability of different strains to rescue the hypoosmotic sensitivity phenotype exhibited by RB562 was assayed by transferring individual worms to 0.8 ml of distilled water and scoring, at 5, 20 and 40 min time points, the phenotype of the worm as either mobile, immobile, rod-like or exploded [72 worms (three replicate *n*=24 assays) per time-point]. The percentages of each phenotype at each time-point are shown graphically (B-D). Strains tested were RB562-derived and harbored extra-chromosomal *fmo-4* or human *FMO4* transgenes that expressed either constitutively [CD1078 (*Pfmo-4::fmo-4::GFP*; B), CD1085 (*Pfmo-4::fmo-4*; B), CD1086 (WRM0636aA04; B) and CD1094 (*Pfmo-4::CeoptHuFMO4*; C)], or were induced following heat-shock [CD1080 (*Phsp-16.2::fmo-4*), CD1081 (*Phsp-16.41::fmo-4*) and CD1089 (*Phsp-16.2::HuFMO4*); D]. Positive (CB1072; B) and negative (CD1079 [pRF4 (*rol-6 su1006*)]; B) control strains were included in some experiments.

DNA sequencing and PCR analysis confirmed RB562 [*fmo-4*(*ok294*)] contains an inactivating deletion of ∼1.5 kb in both copies of *fmo-4* (Fig. S1). When placed in distilled water adult *fmo-4*(*ok294*) hermaphrodites rapidly become immobile, stiff and rod-like before, in many cases, eviscerating violently, usually at the vulva (Fig. 1A). An equivalent phenotype was observed in another *fmo-4* deletion mutant *tm765* (data not shown). The phenotype was not observed with any of four strains lacking functional *fmo-1*, *-2*, *-3* or *-5* (data not shown). The severity and rapid progression of the hypoosmotic sensitivity strongly resembles that of *unc-29*(*e1072*) (Lewis et al., 1980) and we used CB1072 homozygous for this allele as a positive phenotypic control (data not shown and Fig. 1B). *fmo-4*(*ok294*) hypoosmotic sensitivity was phenocopied effectively via *fmo-4* RNAi (Fig. 1A) but not by RNAi targeting *fmo-1*, *-2*, *-3* or *-5* (data not shown). Furthermore, the phenotype was rescued completely in strains (Fig. 1B) carrying either fosmid WRM0636aA04 that contains *fmo-4* located centrally (Fig. S2A), a PCR amplicon (PCR155) encompassing *fmo-4* plus 5′ and 3′ intergenic sequences (Fig. S2A), or fMW002 in which *fmo-4* was tagged, in-frame, with a *gfp* reporter at the C-terminus (Fig. S2C). These data confirmed that the hypoosmotic sensitivity was due solely to loss of functional *fmo-4* with no apparent redundancy with other *fmo* genes. However, rescue was not achieved, even partially, in strains in which transient production of FMO-4 was induced by heat-shock (Fig. 1D). Although we statistically analysed the rescue experiment data (Fig. 1B-D), via ordered logistic regression, the essentially complete rescue observed by transgenic copies of constitutively expressed *fmo-4* meant that in all cases the differences between groups were too extreme for odds ratios to be meaningful.

### FMO-4 and human FMO4 both posses a C-terminal extension

**Fig. 2.**
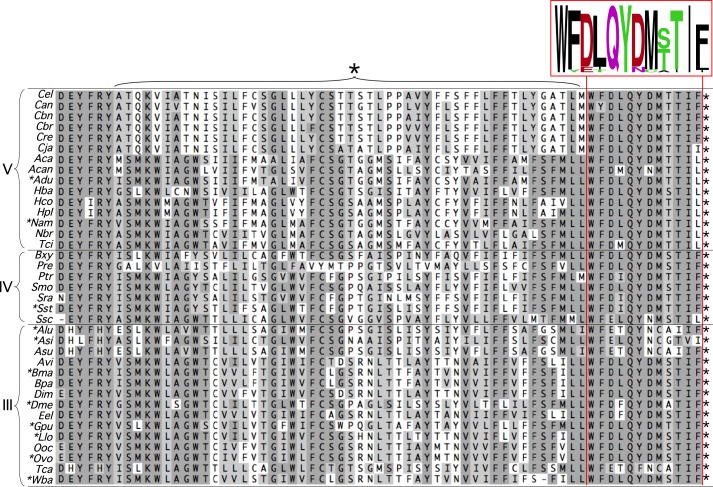
**FMO-4 alignment.** Nematode FMO-4 amino acid sequences were aligned and a region from the aligned C-termini, comprising approximately the last 70 amino acids from each sequence, is shown to illustrate (1) the highly conserved extreme C-termini from within which a consensus motif was derived (red box) (WebLogo; Crooks et al., 2004) and (2) a preceding stretch of essentially conserved length (∼30 residues) but with poor sequence conservation (region marked by *) that is predicted to form two transmembrane helices plus intervening loop. Nematode FMO-4 sequences used to generate the alignment included those from species in clades V [*Caenorhabditis elegans* (*Cel*), *Caenorhabditis angaria* (*Can*), *Caenorhabditis brenneri* (*Cbn*), *Caenorhabditis briggsae* (*Cbr*), *Caenorhabditis remanei* (*Cre*), *Caenorhabditis japonica* (*Cja*), *Ancylostoma caninum* (*Aca*), *Angiostrongylus cantonensis* (*Acan*), *Ancylostoma duodenale* (*Adu*), *Heterorhabditis bacteriophora* (*Hba*), *Haemonchus contortus* (*Hco*), *Haemonchus placei* (*Hpl*), *Necator americanus* (*Nam*), *Nippostrongylus brasiliensis* (*Nbr*), *Teladorsagia circumcincta* (*Tci*)], IV [*Bursaphelenchus xylophilus* (*Bxy*), *Panagrellus redivivus* (*Pre*), *Parastrongyloides trichosuri* (*Ptr*), *Subanguina moxae* (*Smo*), *Strongyloides ratti* (*Sra*), *Strongyloides stercoralis* (*Sst*), *Steinernema scapterisci* (*Ssc*)] and III [*Ascaris lumbricoides* (*Alu*), *Anisakis simplex* (*Asi*), *Ascaris suum* (*Asu*), *Acanthocheilonema viteae* (*Avi*), *Brugia malayi* (*Bma*), *Brugia pahangi* (*Bpa*), *Dirofilaria immitis* (*Dim*), *Dracunculus medinensis* (*Dme*), *Elaeophora elaphi* (*Eel*), *Gongylonema pulchrum* (*Gpu*), *Loa loa* (*Llo*), *Onchocerca ochengi* (*Ooc*), *Onchocerca volvulus* (*Ovo*), *Toxocara canis* (*Tca*) and *Wuchereria bancrofti* (*Wba*)]. No orthologous FMO-4 sequences were identified from Nematoda clades I or II. An asterisk against the species abbreviation indicates a human disease-causing species.

**Fig. 3.**
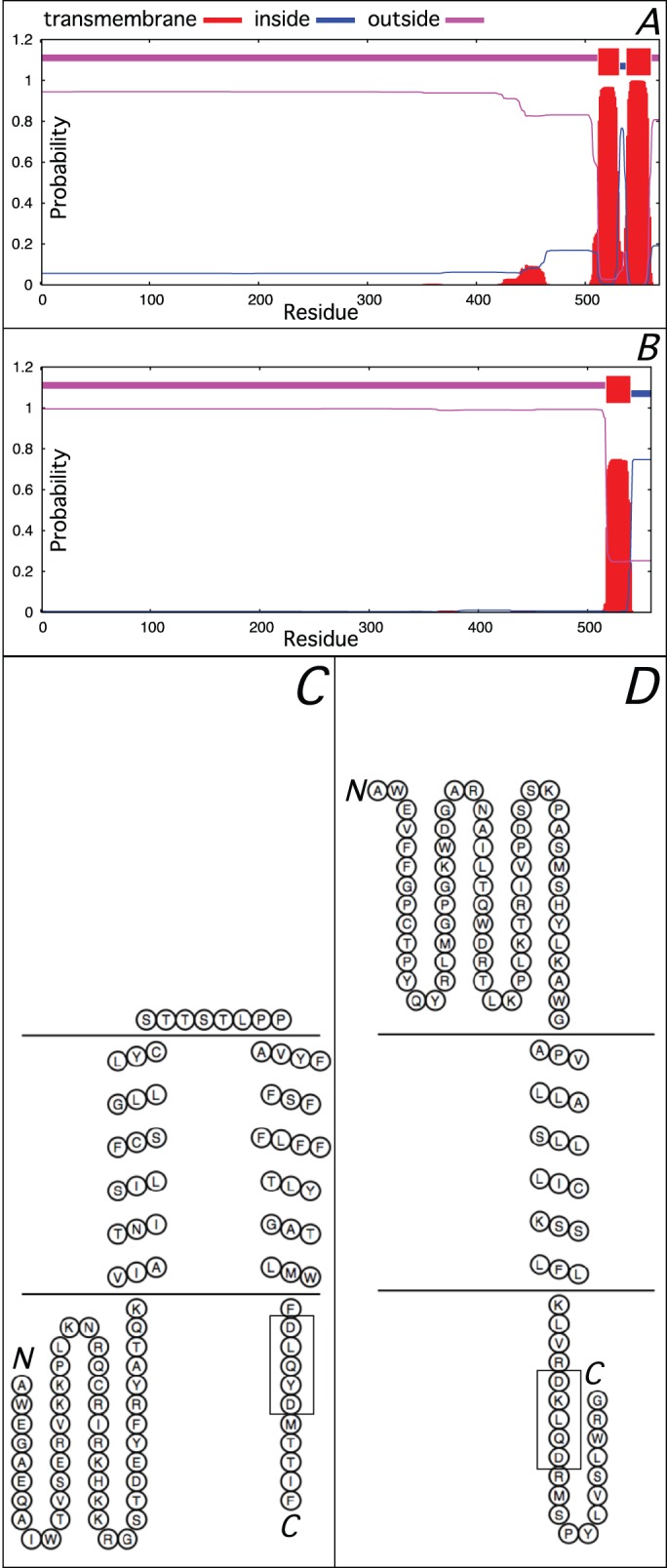
**Predicted transmembrane segments within *C. elegans* FMO-4 and human FMO4.** *C. elegans* FMO-4 (A) and human FMO4 (B) are predicted, with high probability, to contain, respectively, two and one transmembrane segments within their C-termini. Additionally, the extreme C-termini of both *C. elegans* FMO-4 (C) and human FMO4 (D) are predicted to lie outside the membrane lamella and contain, respectively, highly similar DLQYD and DKLQD motifs (boxed). Prediction of trans-membrane segments (A,B) and membrane models (C,D) were generated with TMHMM and HMMTOP, respectively, the latter using the last 100 amino acid residues of the respective protein as input.

*C. elegans* FMO-4 is ∼50 amino acids longer than FMO1, 2, 3 and 5 and these form a distinct C-terminal extension (Fig. S3A). As the presence of this extension differentiates FMO-4 from other nematode FMOs the last 100 residues were used to search protein and genomic DNA databases for orthologous genes. Although *fmo-4* orthologs were apparently absent from Nematoda clades I and II (Blaxter et al., 1998) and also from Annelida and Platyhelminthes, complete or partial FMO-4 amino-acid sequences were retrieved for Nematoda clades III, IV and V and included a number of human disease-causing parasitic species (Fig. 2). Visual examination of a C-terminal section from an alignment of these FMO-4 primary sequences revealed a common stretch of hydrophobic residues followed by highly conserved extreme C-termini from within which a WFDLQYDM(ST)I(FL) consensus was derived (Fig. 2). The conservation of this C-terminal extension prompted us to analyze it for potential secondary structure features. The trans-membrane helix (TMH) tool TMHMM (Krogh et al., 2001), judged to be the most accurate at predicting TMHs (Moller et al., 2001), predicted with high probability (0.95 to 1.0) that, in all cases, the non-conserved hydrophobic region folds into two discrete TMHs, with a loop of 4 to 8 residues, while the final 10 to 12 residues, containing the consensus, lie outside the membrane (Fig. 3A,C).

Designated *FMO2* when first cloned (Dolphin et al., 1992), human *FMO4* (Lawton et al., 1994) encodes a longer FMO due also to the presence of a C-terminal extension (Fig. S3A). In the original report this was proposed to have arisen from a mutational event in an ancestral gene at, or near to, the original stop codon leading to transcriptional run-through to the next in-frame stop triplet. Orthologous *FMO4* genes encoding FMO4s with C-terminal extensions exist in the genomes of many other mammalian species (Burnett et al., 1994 and this study) enabling an alignment of mammalian FMO4s to be compiled using a single species from the Prototheria, Metatheria and each Eutherian order (Fig. S3B). Examination of the aligned C-termini revealed that, apart from the Prototheria and Metatheria FMO4 examples in which it was apparently absent, a conserved motif, [DE]KLQ[DN], could be derived that bears a striking resemblance to a core DLQYD sequence located within the WFDLQYDM(ST)I(FL) consensus of nematode FMO-4 (Fig. S3B). Analysis of the Eutherian FMO4 C-termini predicts each to fold into a single TMH with the [DE]KLQ[DN] motif outside the membrane (Fig. 3B,D and data not shown).

Although the global sequence identity between *C. elegans* FMO-4 and human FMO4 is only 33% (data not shown) it is tempting to propose that the existence in both of a C-terminal extension containing membrane spanning domains and highly similar conserved motifs towards the end is evidence that (1) rather than resulting from a random mutational event the extension evolved in an ancestral gene to serve a clear functional role and (2) nematode *fmo-4* and mammalian *FMO4* evolved from an ancient common ancestor. The two genes would thus be, albeit now highly diverged, potential orthologs. If this is the case then use of the same numerical suffix, decided upon independently for nematode *fmo* (Petalcorin et al., 2005) and mammalian *FMO* (Lawton et al., 1994) nomenclatures, is apposite. However, such coincidental numbering should not be taken to infer orthology between other nematode *fmo* and mammalian *FMO* members.

### FMO4s exist in other species but not all retain C-terminal extensions

Although *FMO4* or *FMO4*-like genes are present in other vertebrate species, not all of these appear to encode proteins that include C-terminal extensions. For example, although BLASTP searches, with the complete human FMO4 sequence as string, identifies putative *FMO4* orthologs in Amphibia, Reptilia and birds none appear to encode a C-terminally extended FMO4 (data not shown). However, a number of *FMO4* genes in the bony fishes do appear to encode proteins extended at their C-termini. Whilst the sequences of the these C-termini varies salmon FMO4 contains the sequence DLLQD that matches closely the [DE]KLQ[DN] consensus in mammalian FMO4 C-termini (Fig. S4).

### Human FMO4 fails to rescue *fmo-4*(*ok294*)

Notwithstanding the relatively low global identity between *C*. *elegans* FMO-4 and human FMO4, we tested the latter for the potential to rescue *fmo-4*(*ok294*). To this end we constructed a fosmid-based transgene in which the complete *fmo-4* CDS was replaced with a synthetic *C*. *elegans*-optimised human FMO4 mini-gene. The transgene failed to rescue the hypoosmotic sensitivity (Fig. 1C). In addition, rescue via heat-shock induced transient expression of human FMO4 was also unsuccessful (Fig. 1D).

### *fmo-4* gene expression analysis

**Fig. 4.**
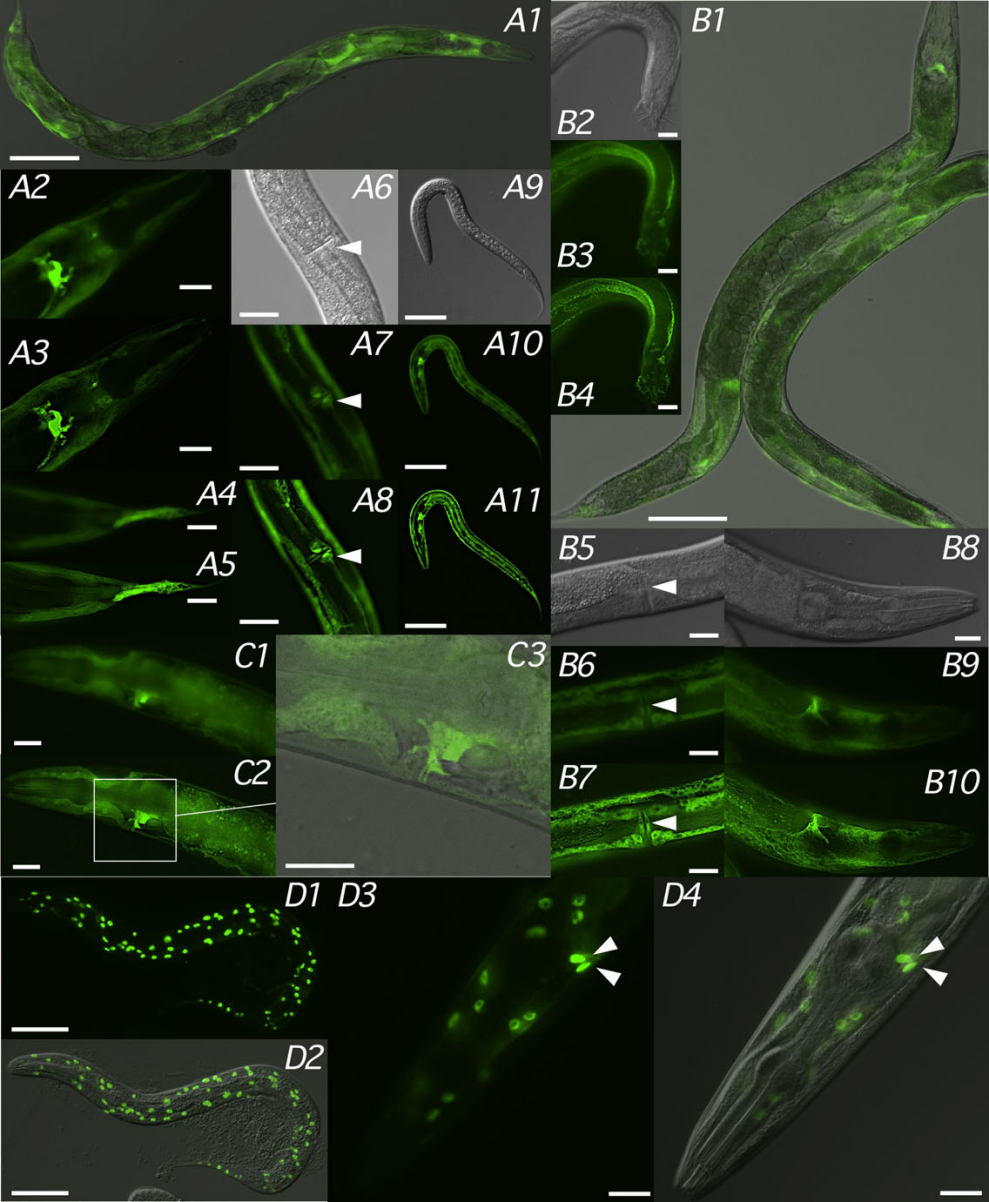
***fmo-4* expression patterns.** Representative gene reporter expression patterns for young adult (A1-A8,B1,B5-B10,C1-C3,D3-D4) or L2/L3 (A9-A11,D1,D2) hermaphrodites (A1-A11,B1,B5-B10,C1-C3,D1-D4) and a single adult male (B2-B4) of strains CD1002 (*Pfmo-4::GFP*; A1-A11), CD1018 (*Pfmo-4::fmo-4::GFP*; B1-B10), BC14787 (*Pfmo-4::GFP*; C1-C3) and CD1090 (*Pfmo-4::NLS::GFP::LacZ*; D1-D4) visualized as a single-channel GFP fluorescence image (A2-A5,A7,A8,A10,A11,B3,B4,B6-B10,D1,D3), the corresponding DIC image (A6,A9,B2,B5,B8) or overlaid (A1,B1,C3,D2,D4). Arrowheads indicate either the vulva (A6-A8,B5-B7) or intense GFP expression localized to duct and pore cell nuclei (D3,D4). Deconvolution (nearest-neighbor) deblurring was applied on some image files (A3,A5,A8,A11,B4,B7,B10) to reveal additional detail. Images were captured at 100× (A1,B1) 200× (A9-A11,D1,D2), 400× (A2-A8,B2-B10,C1-C3,D3,D4) magnification. Scale bars are 100 µm (A1,B1), 50 µm (A9-A11,D1,D2) and 20 µm (A2-A8,B2-B10,C1-C3,D3,D4).

**Fig. 5.**
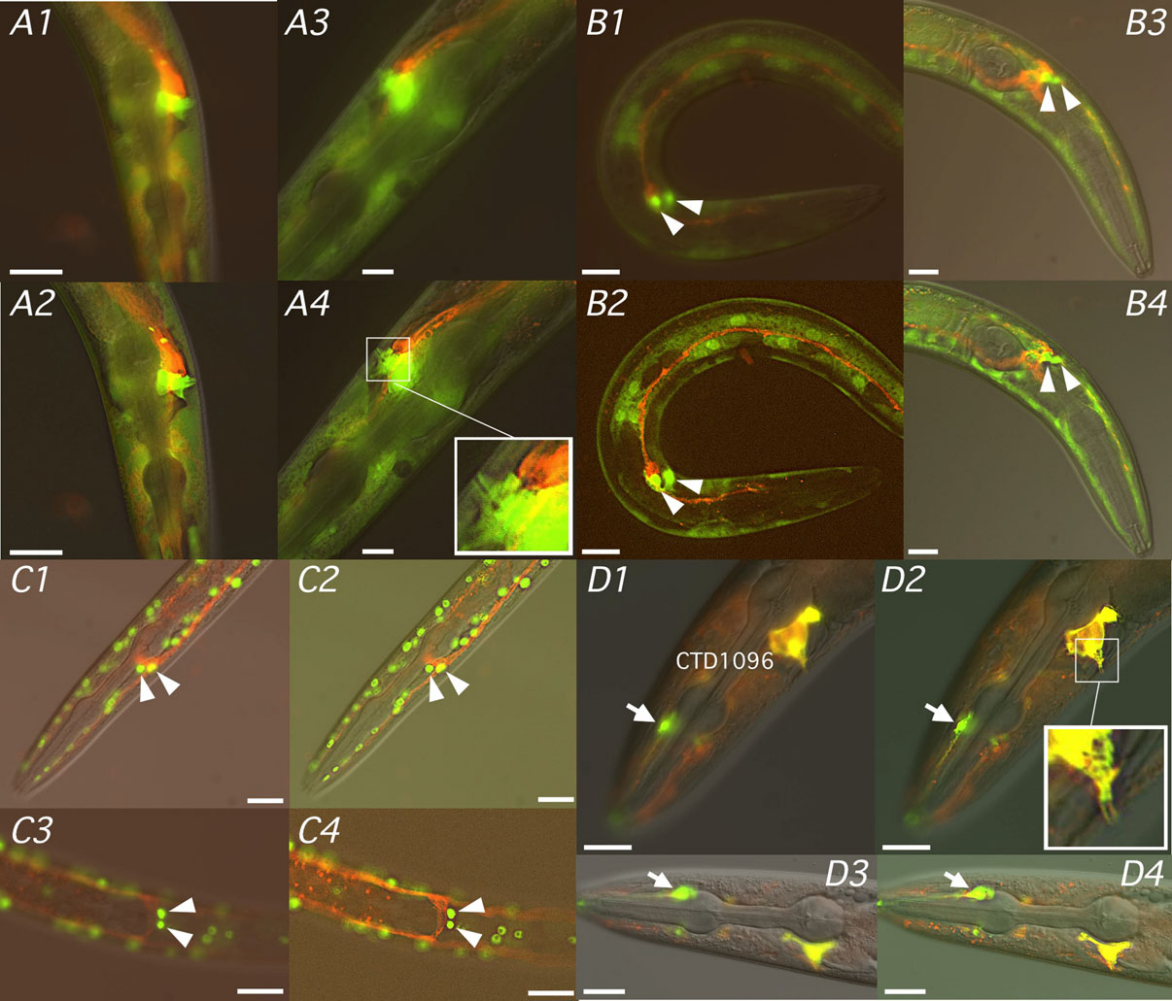
***fmo-4, aqp-8* and *lin-48* co-expression patterns.** Representative reporter gene co-expression patterns for young adult (A1-A4,D1-D4) or L2/L3 (B1-B4,C1-C4) hermaphrodites of strains CD1091, (*Paqp-8::aqp-8::mCherry* ; *Pfmo-4::GFP*; A1-A4), CD1092 (*Paqp-8::aqp-8::mCherry*; *Pfmo-4::NLS::GFP*; B1-B4), CD1093 (*Paqp-8::aqp-8::mCherry*; *Pfmo-4::NLS::gfp::LacZ*; C1-C4) and CD1096 (*Pfmo-4::mCherry* ; *Plin-48::GFP*; D1-D4) visualized as merged green and red fluorescence channels either overlaid with (A1-A4,B3,B4,C1-C4,D1-D4) or without (B1,B2) the corresponding DIC image. Arrowheads indicate intense, punctate GFP expression in duct and pore cell nuclei (B1-B4,C1-C4) while *Plin-48*-driven GFP expression in neuronal support cells is indicated with an arrow (D1-D4). Deconvolution (nearest-neighbor) deblurring of both fluorescence channels was applied to each original file to reveal additional detail (A2,A4,B2,B4,C2,C4,D2,D4). Images were captured at 400× (A1,A2,C1-C4,D1-D4) or 600× (A3,A4,B1-B4) magnification. Scale bars are 20 µm (A1,A2,C1-C4,D1-D4) or 10 µm (A3,A4,B1-B4).

We investigated *fmo-4* expression indirectly in transgenic strains carrying transcriptional and translational reporter constructs (Figs 4, 5). In CD1002, transgenic for pMPFG1 (*Pfmo-4::GFP*) that contains ∼4 kb of *fmo-4* 5′ flanking sequence (Fig. S2B), GFP was expressed diffusely throughout the syncytial hypodermis, including head and tail hyp cells, but was absent from seam cells. Prominent GFP expression was also observed in cells flanking the vulval slit and, most noticeably, in the duct and pore cells just anterior to the posterior bulb of the pharynx (Fig. 4A1-A11). Essentially the same GFP expression was observed in both CD1018, transgenic for the fosmid-based reporter fMW002 (*Pfmo-4::fmo-4::GFP*) (Fig. 4B1-B10), and BC14787 (*rCesF53F4.5::GFP*) (McKay et al., 2003) (Fig. 4C1-C3) in which the PCR-generated *Pfmo-4::GFP* transgene drives *gfp* expression with ∼2.5 kb of upstream sequence (Fig. S2B). GFP expression in the duct and pore cell bodies was clearly visible under higher magnification in all three strains and was also observed in CD1002 and BC14787 outlining the distal section of the excretory duct up to the location of the excretory pore (Fig. 4C2,C3). GFP expression was not observed in the body or canals of the adjacent excretory cell. By targeting GFP to nuclei with a *Pfmo-4::NLS::GFP::LacZ* fusion intense, punctate GFP expression was observed in CD1090 not only in the hypodermal nuclei but also those of the duct and pore cells (Fig. 4D3,D4). The same pattern was observed in *Pfmo-4::NLS::GFP* transgenenic worms except nuclear expression was less punctate (data not shown). These data indicate that, in addition to the hypodermis, *fmo-4* is expressed prominently in the duct and pore cells of the excretory system.

To confirm duct and pore cell expression we generated strains in which an *fmo-4* reporter was coinjected with *aqp-8* (Khan et al., 2013) or *lin-48* (Wang and Chamberlin, 2002) co-markers (Fig. 5) to label the excretory cell and duct and pore cells, respectively. GFP expression in CD1091, which co-expressed *Pfmo-4::GFP* and *Paqp-8::aqp-8::mCherry*, was restricted to hypodermal, duct and pore cells but was absent from the excretory cell which, instead, clearly expressed *Paqp-8*-driven mCherry (Fig. 5A1-A4). In CD1092 (Fig. 5B1-B4) and CD1093 (Fig. 5C1-C4), in which *Pfmo-4::GFP* was replaced with *Pfmo-4::NLS::GFP* and *Pfmo-4::NLS::gfp::LacZ*, respectively, GFP was clearly expressed in the duct and pore cell nuclei located directly anterior to the mCherry-stained excretory cell body. Finally, strain CD1096, that co-expressed *Pfmo-4::mCherry* and *Plin-48::GFP*, was visualized in both green and red channels which, when merged, generated yellow fluorescence confirming co-localization of *fmo-4* and *lin-48* in duct and pore cells (Fig. 5D1-D4).

### Cuticle permeability assay

**Fig. 6.**
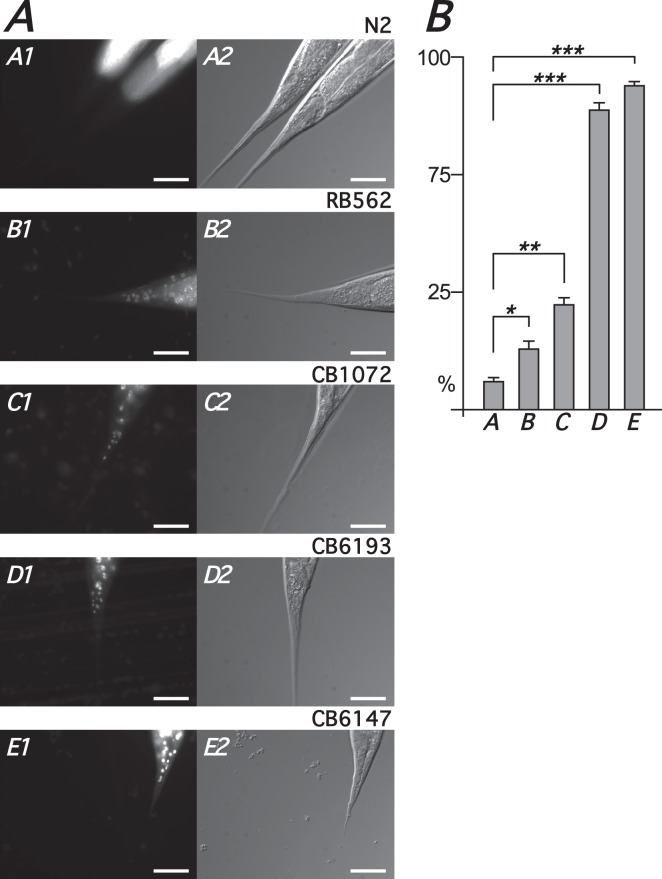
**Comparative cuticle permeability in *fmo-4*, *unc-29* and *bus-8* strains.** (A) Representative DAPI (A1,B1,C1,D1,E1) and corresponding DIC (A2,B2,C2,D2,E2) tail images of N2 (A1,A2), RB562 (*fmo-4*; B1,B2), CB1072 (*unc-29*; C1,C2), CB6147 (*bus-8*; D1,D2), CB6193 (*bus-8*; E1,E2) after incubation with Hoechst 33258. 400× magnification; scale bar=20 µm. (B) Percentage (*y*-axis) of adult animals containing ≥10 distinctly stained nuclei for strains N2 (A), RB562 (B), CB1072 (C), CB6193 (D), CB6147 (E). Error bars represent s.e.m. from four independent replicates (∼50 animals per replicate). *P<0.05, **P<0.01, ***P<0.001.

We examined the integrity of the cuticle/hypodermal barrier in RB562, CB1072 and the epidermis-defective *bus-8* mutants CB6193 and CB6147 (Partridge et al., 2008) with the DNA-binding fluorescent dye Hoechst 33258 (Fig. 6). Stained nuclei were visible in a small percentage of N2 worms (3%, *n*=165) whereas, under the same experimental conditions of incubation and microscopy there was, as expected, extensive and highly statistically significant nuclear staining in CB6193 (84%, *n*=193, *P*<0.001) and CB6147 (89%, *n*=197, *P*<0.001) (Fig. 6D-E). Although significant compared to N2 far fewer nuclei were unambiguously stained in RB562 (15%, *n*=184, *P*<0.05) and CB1072 (22%, *n*=193, *P*<0.01) in comparison to the extensive and distinct staining in the *bus-8* mutants (Fig. 6B,C).

### FMO-4 exhibits S-oxygenase activity

**Fig. 7.**
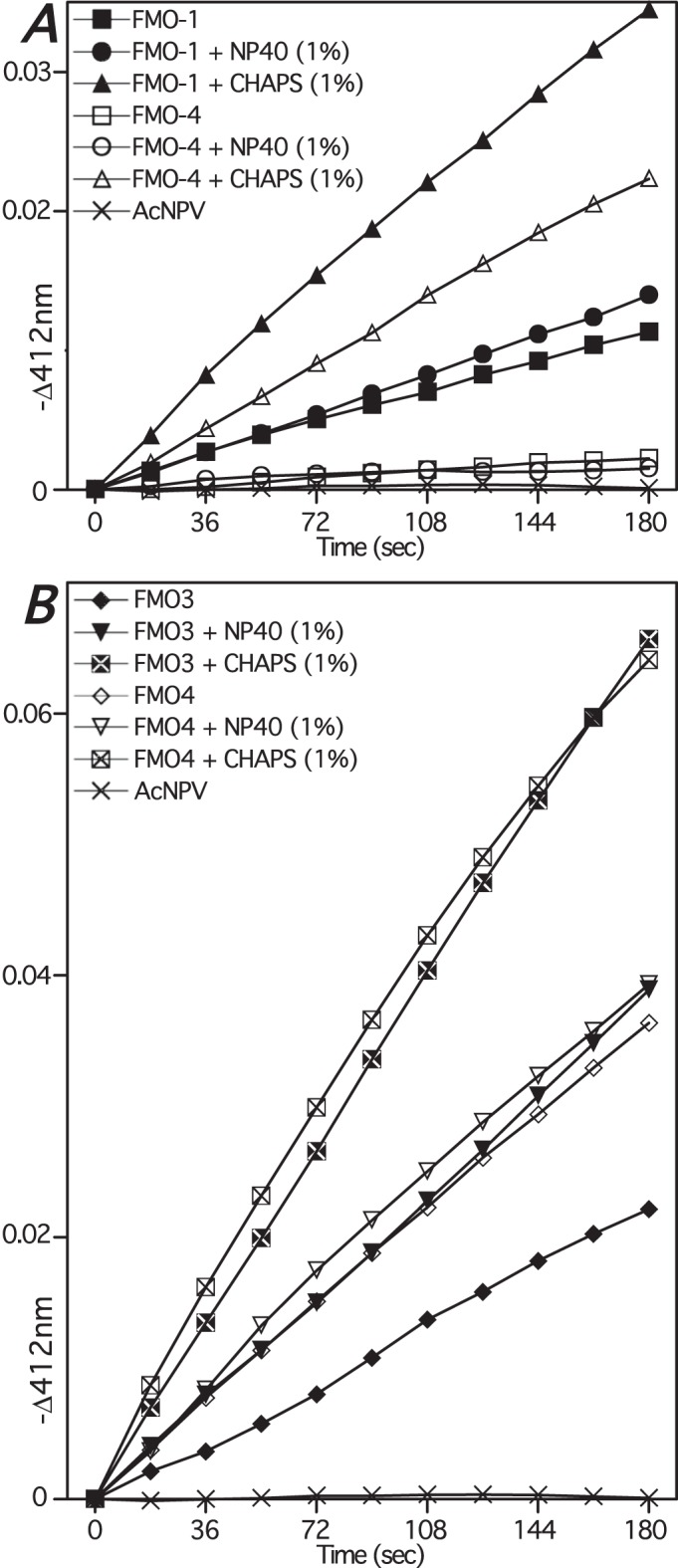
**Functional characterization of heterologously expressed *C. elegans* and human FMOs.** Methimazole oxidation-dependent nitro-5-thiobenzoate oxidation, monitored indirectly as the time-dependent difference in absorbance at 412 nm, was catalyzed via microsomal membrane protein isolated from *Sf9* cells infected with baculoviruses vCD020 (950 µg, A), vCD024 (930 µg, A), vCD030 (330 µg, B) or vFMO3 (315 µg, B) encoding, respectively, *C. elegans* FMO-1, *C. elegans* FMO-4, human FMO4 and human FMO3. Reactions contained microsomal protein resuspended in Tricine buffer (pH 8.4) alone or with additional NP40 or CHAPS detergent (1% v/v).

Preliminary functional characterization of *C. elegans* FMO-1 and FMO-4 plus human FMO3 and FMO4, produced via heterologous expression in insect cells, was undertaken. As specific antibodies against FMO-1 and FMO-4 were unavailable we chose to bypass examination of microsomal protein by immunoblotting and move directly to investigate the ability of membranes to catalyze S-oxidation of the archetypal mammalian FMO substrate methimazole (Dixit and Roche, 1984) as described (Dolphin et al., 1997, 1998). Membranes from cells infected with vCD020 (*C. elegans* FMO-1) or vCD024 (*C. elegans* FMO-4) catalyzed S-oxidation with essentially maximal specific activities of, respectively, 0.02 and 0.005 nmol of methimazole oxide formed/min/mg microsomal protein (Fig. 7A). Although, the non-ionic detergent NP-40 had little effect on the activity of either FMO addition of the zwitterionic CHAPS increased FMO-1 and FMO-4 activities to 0.06 and 0.05 nmol/min/mg microsomal protein, respectively (Fig. 7A). We also expressed human FMO3 and FMO4 – the former as a positive control as we have expressed and characterized FMO3 previously (Dolphin et al., 1997) and the latter because of the putative relationship with FMO-4. Initial maximal S-oxidation rates with membranes from cultures infected with vFMO3 (human FMO3) or vCD030 (human FMO4) were, respectively, 0.15 and 0.22 nmol/min/mg microsomal protein (Fig. 7B). NP40 had little effect on the base activity of FMO4-catalyzed methimazole oxidation whereas the FMO3 activity was increased to 0.22 nmol/min/mg microsomal protein (Fig. 7B). Again, addition of CHAPS had a clear stimulatory effect and both human FMO activities were increased to ∼0.4 nmol/min/mg microsomal protein (Fig. 7B). The base methimazole oxidation activity with FMO3-containing microsomes is less than we reported previously (2.0 nmol/min/mg microsomal protein) (Dolphin et al., 1997) and this likely reflects less than optimal expression conditions and/or microsomal membrane isolation resulting in lower specific yields of FMO. This may also explain the low activities observed with both recombinant worm FMOs. That said, the preliminary experiments reported here confirm both worm FMOs are indeed able to catalyze methimazole S-oxidation.

## DISCUSSION

Loss of *fmo-4* had no apparent effect upon the ability of *C. elegans* to osmoregulate under laboratory conditions as there was no evidence of compromised water homeostasis, such as fluid accumulation or swelling, during passage on NGM plates or when transferred to M9 buffer (data not shown). However, following transfer to distilled water loss of *fmo-4* results in dramatic hypoosmotic hypersensitivity – worms are unable to prevent overwhelming water influx, swell and, in many cases, rupture violently due to the high internal turgor pressure. The phenotype was observed in two independent *fmo-4* deletion strains, was phenocopied by RNAi and rescued completely with extrachromosomal *fmo-4*. These data confirm that the inability to osmoregulate under sudden hypotonic stress, as would likely be experienced by *C. elegans* in its natural habitat following rainfall, is due solely to loss of functional *fmo-4*. Rescue was not achieved when transgenic *fmo-4* was induced following heat shock perhaps because this did not result in sufficient enzyme being synthesized to provide adequate protection from subsequent osmotic challenge. The *fmo-4* expression pattern (Figs 4,5), investigated here in some detail, supports such a role as reporter expression was detected in the hypodermis and duct and pore cells all of which are involved in water balance control (Choe, 2013). Expression was especially prominent in duct and pore cells but, interestingly, was absent from the adjacent excretory cell. Laser ablation of any one of these three cells leads to dysfunctional water balance under isotonic conditions and, when osmotically challenged with acute hypotonicity, a more severe hypoosmotic sensitivity resembling that of *fmo-4*(*ok294*) (Nelson and Riddle, 1984).

Although we did not investigate it here loss of FMO-4, whether via deletion (Shen et al., 2005) or RNAi (Rual et al., 2004), has also been associated with reduced viability, e.g. the former report described an ∼60% reduction in brood size that was further reduced under hypoxia. Whether the embryonic lethality under normoxia is associated with the compromised osmoregulatory competence we report here is not clear. Interestingly, null mutations in *rdy-2*, a gene encoding a novel tetraspan protein, are associated with osmoregulatory defects and lethality as larvae die with a fluid-filled and rod-like phenotype (Liegeois et al., 2007). Intriguingly, although there is no evidence that *fmo-4* and *rdy-2* are located together in an operon, they are adjacent and on the same strand on chromosome V.

FMOs are classified conventionally as XMEs that detoxify and, occasionally, bioactivate diverse xenobiotics (Ziegler, 2002; Krueger and Williams, 2005). However, individual plant and animal, including human, FMOs have now been identified or implicated in a range of physiological or metabolic activities in many cases mediated via the oxygenation of a specific endogenous substrate. Although FMO-4 may still function in xenobiotic detoxification the clear involvement of FMO-4 in water balance suggests a major role is one of endogenous osmoregulation. Although it is not clear at this stage how this role is enzymatically accomplished we speculate below on possible mechanisms.

There was evidence for at least partial loss of epidermal barrier function in *fmo-4*(*ok294*) as dye uptake studies revealed a small but significant increase in nuclear staining compared to N2. The degree of staining was, however, far less than that observed in two *bus-8* mutants that exhibit clear disorganized body wall structures reflecting loss of a glycosyltransferase activity required for epidermis morphogenesis (Partridge et al., 2008). We did not observe any gross epidermal or cuticle defects in adult RB562 (data not shown). Interestingly, the single FMO of yeast promotes protein disulphide bond formation in the ER by generating permissive oxidative conditions via biological thiol oxidation (Suh et al., 1999). Thus, it is conceivable that, were FMO-4 to have the same activity, hypodermally-expressed *FMO-4* may contribute to cuticular collagen disulphide bond formation by a similar mechanism.

FMOs classically catalyze heteroatom oxidation via the C(4a)hydroperoxide flavin derivative. There are, however, reports of FMOs catalyzing Baeyer–Villiger oxidations which, by inserting oxygen into the C-C bond adjacent to a carbonyl, convert ketones to esters and cyclic ketones to lactones (Chen et al., 1995; Lai et al., 2011; Meng et al., 2015). Thus, unless FMO-4 has a yet-to-be recorded enzymatic activity the osmoregulatory role is likely mediated either via heteroatom oxygenation or perhaps even Baeyer–Villiger oxidation. There is, however, one other perhaps less likely mechanism – generation of H_2_O_2_. Considering this option first, heterologously expressed mammalian FMO can generate H_2_O_2_ not only during enzymatic uncoupling but also with substrate present and this NADPH oxidase activity can result in not insubstantial H_2_O_2_ production (Siddens et al., 2014). H_2_O_2_ and other reactive oxygen species increase rapidly in a number of mammalian cell lines following hypotonic exposure (Hoffmann et al., 2009), including NIH3T3 fibroblasts (Lambert, 2003) and hepatocytes (Varela et al., 2004), and activate or potentiate swell-induced osmolyte and ion efflux channels. Thus, it is feasible FMO-4 might reduce hypotonicity-induced water influx via FMO-4-generated H_2_O_2_-mediated transporter activation.

Most likely, however, FMO-4 acts via classic heteroatom monooxygenation in an endogenous substrate and the resulting product acts to attenuate water accumulation under hypoosmotic conditions. Such a product might be generated in response to water influx or, alternatively, generated constitutively and sequestered and thus be available for immediate use under hypotonic stress. The product would likely be electroneutral and tolerated at high concentration to avoid disturbance of intracellular ionic and protein homeostasis – characteristics typical of osmolytes (Yancey et al., 1982). The product remains to be identified but glycerol, the osmolyte accumulated in *C. elegans* under hyperosmotic stress, can be excluded as it is synthesized by glycerol-3-phosphate dehydrogenase (Lamitina et al., 2004). Although trimethylamine N-oxide (TMAO), an osmolyte utilized by many marine species (Yancey et al., 1982) and generated via FMO (Lang et al., 1998), is a possible candidate it is not considered likely as, if this were the case, *C. elegans* would be commonly associated with a fish odour as TMAO is readily reduced to the malodorous, volatile base.

Setting identification to one side how might this product act in hypotonicity? One possibility is that, stored in the hypodermis as an osmolyte, it would, during hypotonicity-induced water influx, be available for efflux via transporter systems thereby reducing the osmotic gradient and water ingress. Another, potentially compatible model can be built from the observation that FMO-4 is expressed prominently in duct and pore cells but is absent from the excretory cell. Assuming the putative FMO-4 product is concentrated in these two cells this could establish a strong osmotic gradient from excretory cell to duct and pore cells encouraging water movement down the gradient through the excretory system exiting at the excretory pore. The presence of aquaporin channels in the excretory cell (Huang et al., 2007) would be available to transfer water, drawn from the hypodermis and/or pseudocoelom, into the excretory cell canals before passing into the excretory duct at the secretory-excretory junction. If this osmotic gradient is compromised in *fmo-4*(*ok294*) worms water flow rate may be insufficient to prevent swelling and rupture following acute hypotonic exposure. There are, of course, limitations with this model – how, for example, would excess water be encouraged to remain in, and move down, the duct lumen? One possibility is that the cuticle surrounding the duct is less water permeable than cuticle elsewhere. Also, Nelson and Riddle (1984) reported that, although muscle-like filaments were not observed in duct or pore cells, the excretory duct pulsates in dauer larvae resulting in fluid exiting at the pore. Pulsation has also been observed in excretory ducts of larval stages of other nematodes (Atkinson and Onwuliri, 1981). Thus, it is possible that similar, transient pulsations might occur in the adult excretory duct under conditions of excess water influx. Another potential complication is the reported presence of gap junctions between excretory, duct and pore cells (Nelson et al., 1983) as these would presumably permit diffusion of any FMO-4 product thus disrupting the proposed osmotic gradient. Interestingly, a more recent report (Altun et al., 2009) failed to detect expression of gap junction-associated innexin proteins in duct or pore cells suggesting such connections may not exist.

Visual examination of aligned FMO-4 sequences revealed a shared C-terminal extension comprising a stretch of hydrophobic residues, predicted to fold into two TMHs, and ending in a core DLQYD motif. This sequence, which was predicted to lie outside the membrane, is not likely to be involved in formation or anchoring of the adjacent TMH as residues that commonly flank and help stabilize a TMH, such as Tyr, Trp, Arg and Lys, are rarely conserved (G. von Heijne, Stockholm University, personal communication). Mammalian FMO2 and 4, when expressed heterologously, can catalytically tolerate a loss of ∼30 residues from the C-termini (Lawton and Philpot, 1993; Itagaki et al., 1996) suggesting the FMO-4 C-terminus may also not be required for enzymatic activity. Thus, the consensus-containing extreme C-terminal sequence likely performs a distinct functional task perhaps related to osmoregulation. Interestingly, examples of osmosensing via a C-terminal protein region exist, most notably the regulatory domain of the glycine-betaine uptake system BetP in the bacterium *Corynebacterium glutamicum* (Kramer, 2009).

Mammalian FMO4s are also extended at their C-termini many of which contain a [DE]KLQ[DN] motif that bears significant similarity with the FMO-4 C-terminal core DLQYD consensus. The preceding FMO4 sequence is also predicted to span the membrane albeit only once which, in comparison to FMO-4, would result in C-terminal tail exiting the opposite side of the membrane. While the global identity between *C. elegans* FMO-4 and human FMO4 is relatively low the presence in both of C-terminal extensions that contain predicted TMH domain(s) and conserved, highly similar sequence motifs suggest to us that nematode *fmo-4* and mammalian *FMO4* could have evolved from a common, albeit ancient ancestor making them potential orthologs. Because this putative phylogenetic relationship would also suggest potential functional homology we investigated whether human FMO4 could rescue the *fmo-4*(*ok294*) osmoregulatory defect. However, neither constitutive nor transient expression was successful. Assuming human FMO4 was expressed successfully these results suggest that human FMO4 and *C. elegans* FMO-4 do not functionally overlap; perhaps not surprising considering the relatively low shared global sequence identity.

As part of this study we also undertook preliminary characterization of *C. elegans* FMO-1 and FMO-4. Both enzymes exhibited S-oxidation activity with the FMO probe substrate methimazole that was, in both cases, stimulated by addition of detergents. Unfortunately, lack of expressed protein and time precluded further investigations into additional detergent effects or other potential activity modulators nor were we able to examine N-oxidation. We also expressed human FMO3 and FMO4 – the former as a positive control and the latter because of the putative relationship with FMO-4. FMO4 was expressed successfully as a full-length protein containing the C-terminal extension sequence. Previous attempts to express FMO4 as a functional enzyme in yeast or bacteria were only successful when this region was deleted (Itagaki et al., 1996). Future work will explore the expression and functional characteristics of FMO-4 and FMO4 as C-terminally truncated proteins.

Although the specific catalytic activity underlying the osmoregulatory role of FMO-4 has yet to be determined it would be theoretically possible to screen a series of candidate substrates with heterologously expressed enzyme. An alternative and non-biased approach would be to employ a metabonomics-LC-MS-based system to interrogate *C. elegans* extracts following incubation with recombinant FMO-4 (de Carvalho et al., 2010; Prosser et al., 2014). This approach has been used successfully to identify endogenous ligands for a number of enzymes (Saghatelian et al., 2004; Saito et al., 2006). By combining the approach with ^18^O_2_ labeling the method has also been applied to deorphanize human cytochrome P450 enzymes (Tang et al., 2009) – a strategy that could also be applied to identify endogenous FMO-4 ligand(s).

The increasing number of reports describing FMO involvement in endogenous activities, including life span (Leiser et al., 2015), energy balance (Veeravalli et al., 2014), metabolic ageing (Gonzalez-Malagon et al., 2015) and even atherogenesis and cholesterol metabolism (Schugar and Brown, 2015), illustrate these XMEs also have, perhaps in many cases, specific biological roles. We have now added another entry to this list by describing an osmoregulatory role for *C. elegans* FMO-4. Sequence features shared between *C. elegans* FMO-4 and mammalian FMO4 suggest the corresponding genes may have evolved from a common, ancient ancestor. If this reflects a shared, or once shared, functional role then is it possible FMO4 may also have, or once had, an osmoregulatory function. Interestingly, human FMO4 expression, whether measured via mRNA (Yanai et al., 2005; Nishimura and Naito, 2006; Zhang and Cashman, 2006; Dezso et al., 2008) or protein (Novick et al., 2009) abundance, reveals that while constitutively expressed in many tissues human FMO4 is often most abundantly expressed in the kidney, a site that would certainly appear to support such a proposal.

## MATERIALS AND METHODS

### General *C. elegans* and molecular biological methods

Worm maintenance was performed as described (Sulston and Hodgkin, 1988) under approved conditions. RNAi by feeding was performed essentially as described (Timmons and Fire, 1998). Construct fidelity was confirmed by sequencing. Strains carrying heat shock-inducible transgenes were heat shocked (32°C, 4 h) and recovered (20°C, 2 h). Counter-selection recombineering of *C. elegans* fosmid clones in *E. coli* MW005 (Westenberg et al., 2010) using a *rpsL-tet(A)* selection cassette (RT-cassette), amplified from pBAC-RT (Stavropoulos and Strathdee, 2001), was performed as described (Dolphin and Hope, 2006; Hirani et al., 2013). Cloning strategies and sequence analyses were performed with MacVector (MacVector Inc). For creation of transgenic lines young adult hermaphrodites were co-injected, into the syncytial gonad, with a mixture of plasmid and/or fosmid DNA (10-50 µg/ml) and, when required, markers pRF4 [*rol-6* (*su1006*)], P*myo-2::gfp*, P*myo-3::gfp* or P*ofm-1::gfp* (5-50 µg/ml). pBluescript and/or sheared salmon-sperm DNA were included to increase injected DNA concentration to ∼100 µg/ml. For both gene expression and rescue experiments at least two transgenic lines were investigated.

### *C. elegans* strains

Strains N2, RB671 [*fmo-1*(*ok405*) *IV*], VC1668 [*fmo-2*(*ok2147*) *IV*], RB686 [*fmo-3*(*ok354*) *III*], RB562 [*fmo-4*(*ok294*) *V*], CB1072 [*unc-29*(*e1072*) *I*], BC14787 [dpy-5(e907) *I*;[*rCesF53F4.5::GFP+pCeh361*]] and CB6193 [*bus-8*(*e2885*) *X*] were obtained from the Caenorhabditis Genetics Centre. CB6147 [*bus-8*(*e2882*) *X*] was a gift of J. Hodgkin, University of Oxford. *tm765* and *tm2438* were from the National Bioresource Project, Japan. RB562 was backcrossed (6×) to N2 using confirmatory multiplex (ODNs 4517, 4518 and 4519; Fig. S1, Table S1) single-worm PCR. In addition, ODN 4565 (Table S1), by annealing across the *ok294* deletion point, was used with ODN 4368 (Table S1) to selectively amplify from worms containing *ok294* (Fig. S1). Fidelity of transgenic strains created as part of this study (Table S2) carrying either gene expression or rescue extra-chromosomal constructs was confirmed by expression of the transformation marker or, where appropriate, single-worm PCR.

### RNAi constructs

RNAi plasmids pMPK2R2, pMPK5R1, pMPYR1, pMPFR1 and pMPHR1 for *fmo-1* to *-5*, respectively, were built as described (Petalcorin et al., 2005). Additional *fmo*-*1*, -*2*, -*4* and -*5* RNAi plasmids with respective WormBase IDs WBRNAi00016779, WBRNAi00016781, WBRNAi00015527 and WBRNAi00016311 were as described (Kamath et al., 2003).

### Rescue constructs

Three constructs were built to investigate whether heat shock-induced FMO-4 or human FMO4 could rescue *fmo-4*(*ok294*). pMPFNA1 was constructed by inserting, via *Kpn*I and *Sac*I, the FMO-4 CDS amplified from cDNA (ODNs fmo-4F and fmo-4R; Table S1), into pPD49.83 (Fire et al., 1990). pCD037 was derived by transferring the FMO-4 CDS from pMPFNA1 into the equivalent sites within pPD49.78. pCD070 was generated by inserting a human FMO4 CDS, excised from clone 13W (Dolphin et al., 1992) using *Nco*I-*Pvu*II, into pPD49.78 via *Nco*I-*Eco*RV.

A fosmid-based *Pfmo-4*::*FMO4* construct was recombineered by replacing the *fmo-4* gene sequence in WRM0636aA04 (Fig. S2A) with a PCR-amplified RT-cassette (ODNs 4442 and 4443; Table S1) to create fCD010 and then swapping out the RT-cassette with a 2.3 kb synthetic (Genewiz) *C. elegans* codon-optimized human *FMO4* mini-gene, PCR-amplified from the commercially provided plasmid (ODNs 4740 and 4741; Table S1), to create fCD012 (Fig. S2D). Gene finder Augustus (http://augustus.gobics.de/) predicted splicing fidelity of native and artificial introns in the *FMO4* mini-gene sequence at locations equivalent or close to those in *fmo-4*.

In addition to the plasmid and fosmid-based constructs, a PCR amplicon, PCR155, was generated by amplifying (ODNs 4551 and 4552; Table S1) a 7.5 kb region from WRM0636aA04 containing *fmo-4* plus 5′ and 3′ flanking regions up to the respective flanking genes *F53F4.22* and *F53F4.19* (Fig. S2A).

### Gene expression reporter constructs

In addition to pMPFG1 (P*fmo-4::gfp*) (Petalcorin et al., 2005) a number of other plasmid- and fosmid-based *fmo-4* reporter constructs were generated (Fig. S2). pCD071 (*Pfmo-4::NLS::gfp::LacZ*) was generated by replacing *LacZ* in pMPFL1 (Petalcorin et al., 2005) with *NLS::gfp::LacZ* excised from pPD96.02 (Fire et al., 1990) with *Sac*I. Subsequently, pCD073 (*Pfmo-4::NLS::gfp*) was generated by replacing the *NLS::gfp::LacZ-unc-54* sequence in pCD071 with a *NLS::gfp::unc-54* fragment excised from pPD104.53 (Fire et al., 1990) with *Sac*I and *Spe*I. pCD078 (*Pfmo-4::mCherry*) was built by replacing *gfp* in pMPFG1, excised with *Kpn*I and *Apa*I, with mCherry CDS, PCR-amplified from pNH077 (Hirani et al., 2013) (ODNs 4756 and 4757; Table S1), via the same sites. The fosmid-based translational reporter fMW002 (*Pfmo-4::fmo-4::gfp*) was recombineered by, first, inserting a PCR-amplified RT-cassette (ODNs 4357 and 4358; Table S1) six triplets 5′ of the *fmo-4* stop codon in WRM0636aA04 generating fMW001, then replacing the RT-cassette with a *gfp* CDS (minus stop), PCR-amplified from pPD95.77 (Fire et al., 1990) (ODNs 4359 and 4360; Table S1), to generate fMW002 in which *Pfmo-4::fmo-4::gfp* is flanked by ∼19 kb and 10 kb of 5′ and 3′ genomic sequence, respectively (Fig. S2C).

### Bioinformatics

Nematode FMO-4 sequences were obtained either directly from WormBase (WS242) or via BLASTP or TBLASTN searches with the last 100 amino acids of *C. elegans* FMO-4 as string. Amino acid sequence alignments were generated with ClustalW and optimized ‘by eye’. Transmembrane predictions were performed with TMHMM (Krogh et al., 2001) and membrane models generated with HMMTOP (Tusnady and Simon, 2001).

### Hypoosmotic sensitivity assay

For each strain 24 individual 5-7 day adult hermaphrodites were transferred to distilled water (1 ml in each of 24 wells of a 48-well plate) and each worm scored at 5 min, 20 min and 40 min as either mobile, immobile, rod-like or exploded. Each assay was performed in triplicate and proportions calculated for each phenotype at each time point. Ordered logistic regression (Long and Freese, 2014) was performed by investigating three outcomes: time to worm becoming immobile, rodlike or exploded; each with four possible values (≤5 min, 5-20 min, 20-40 min, and ≥40 min), corresponding to time when first seen in the condition (5 min, 20 min, 40 min and Never) and results expressed as odds ratios. Formal statistical significance was set at *P*<0.05. Data was analysed in Stata (version 13.1).

### Cuticle barrier assay

Synchronized young adult worms were incubated (15 min, room temperature) in M9 buffer containing gelatin (0.1% w/v) and Hoechst 33258 (10 µg/ml), washed (M9 plus gelatin) and imaged (DAPI). For each strain four independent stainings were performed (*n*=∼50 worms/staining). Worms were scored, under the same conditions of incubation and microscopy, as ‘stained’ if ≥10 nuclei were clearly visible under DAPI imaging. Strains were compared against N2 using a two-tailed *t*-test on arcsin-transformed data.

### Construction of recombinant baculoviruses

pFastBac1-based shuttle vectors pCD020, pCD024 and pCD075, containing FMO-1, FMO-4 and human FMO4 CDSs, respectively, were constructed as follows. pCD020 was built by inserting a FMO1 CDS PCR-amplified (ODNs 4458 and 4459; Table S1) from pMPK2B1 (Petalcorin et al., 2005), into pFastBac1 via *Bam*HI and *Pst*I. To build pCD024 a FMO-4 CDS, PCR-amplified from pMPFNA1 (ODNs 4464 and 4465; Table S1), was inserted into pFastBac1 via *Bam*HI and *Pst*I. pCD075 was constructed by replacing a human FMO2 CDS in pFastFMO2/2/16 (Dolphin et al., 1998) with a human FMO4 CDS excised from clone 13W (Dolphin et al., 1992). Bacmid DNAs, bCD020, bCD024, bCD030 and bFMO3, generated via site-specific transposition (Bac-to-Bac system, Thermo Fisher) from the respective shuttle vectors pCD020, pCD024, pCD075 and pFastFMO3 (Dolphin et al., 1997), were transfected into *Sf9* insect cells and, following incubation (28°C, 72 h) and cell pelleting, the resulting respective baculoviruses vCD020 (*C. elegans* FMO-1), vCD024 (*C. elegans* FMO-4), vCD030 (human FMO4) and vFMO3 (human FMO3) were collected in the filtered (0.2 µm) supernatant and stored (4°C).

### Baculovirus-mediated expression and preparation of microsomal membrane vesicles

*Sf*9 cells were passaged using Sf-900III (Thermo Fisher) media containing penicillin-G (100 units/ml) and streptomycin sulphate (0.1 mg/ml). Virus was amplified using a multiplicity of infection (MOI) of ≤0.1. For expression, *Sf*9 cells [200 ml, 10^6^ cells/ml, 1 litre Spinner flask (Bellco)] were infected (MOI 10) and incubated (72 h, 100 rpm, 28°C) in Sf-900III supplemented with FAD (10 µg/ml). Cells were pelleted, resuspended (0.154 M KCl, 50 mM Tris pH 7.4, 0.2 mM PMSF) and lysed by sonication. Debris was removed by centrifugation (10,000 ***g***, 15 min, 4°C) and microsomal membrane vesicles were pelleted from the resulting supernatant (100,000 ***g***, 1 h, 4°C), resuspended [0.154 M KCl, 10 mM HEPES pH 7.5, 1 mM EDTA, 20% (v/v) glycerol] and aliquots stored (−80°C). Protein concentration was determined by BCA assay.

### FMO assay

FMO activity, determined via methimazole oxidation-dependent nitro-5-thiobenzoate oxidation monitored as the time-dependent difference in absorbance at 412 nm (Dixit and Roche, 1984), was performed as described (Dolphin et al., 1997, 1998). When used, detergents NP40 or CHAPS were added directly to both sample and reference cuvettes (1% v/v).

### Microscopy

DIC and fluorescence images were observed on an Olympus BX61 upright microscope equipped with an F-ViewII camera and processed (cellSens Dimension software).
