# *C. elegans* flavin-containing monooxygenase-4 is essential for osmoregulation in hypotonic stress

**DOI:** 10.1242/bio.019166

**Published:** 2016-04-27

**Authors:** Nisha Hirani, Marcel Westenberg, Paul T. Seed, Mark I. R. Petalcorin, Colin T. Dolphin

There was an error in the Biology Open article doi:10.1242/bio.017400 published ahead of print on 23 March 2016.

The author list was incomplete, and the text highlighted in bold below has been changed in the author list, the author affiliations and the author contribution sections:

Nisha Hirani^1,^*, Marcel Westenberg^1,‡^, Paul T. Seed^2^, **Mark I.R. Petalcorin^1,§^** and Colin T. Dolphin^1,¶^

^1^Institute of Pharmaceutical Science, King's College London, Franklin-Wilkins Building, 150 Stamford Street, London SE1 9NH, UK. ^2^Division of Women's Health, King's College London, St Thomas’ Hospital, London SE1 7EH, UK. *Present address: The Francis Crick Institute, Lincoln's Inn Fields Laboratories, 44 Lincoln's Inn Fields, London WC2A 3LY, UK. ^‡^Present address: Dutch National Plant Protection Organization (NPPO-NL), National Reference Centre, P.O. Box 9102, Wageningen 6700 HC, The Netherlands. **^§^Present address: PAPRSB Institute of Health Sciences, Universiti Brunei Darussalam, Jalan Tungku Link, BE1410, Brunei Darussalam.**

^¶^Author for correspondence (colin.dolphin@kcl.ac.uk)

**Author contributions**

**N.H., M.W. and M.I.R.P.** undertook the generation of constructs, *C. elegans* husbandry, molecular genetic and phenotype analysis studies and microscopy. P.T.S. performed the statistical analysis. C.T.D. designed the experiments, participated in the experimental work and wrote the manuscript.

Biology Open and the authors apologise to the readers for any confusion that this omission might have caused.

